# Inappropriate Medication Use in Hospitalized Patients Diagnosed with Parkinson’s Disease

**DOI:** 10.3390/pharmacy6030100

**Published:** 2018-09-15

**Authors:** Nicholas Cox, Jessica M. Louie, Benson H. Sederholm

**Affiliations:** 1 Department of Pharmacotherapy, University of Utah College of Pharmacy, 30 S. 2000 E., Salt Lake City, UT 84112, USA; 2 Department of Pharmacy Practice, West Coast University School of Pharmacy, Los Angeles, UT 90004, USA; jlouie@westcoastuniversity.edu; 3 Department of Pharmacy Services, University of Utah Health, 50 N. Medical Drive, Salt Lake City, UT 84132, USA; benson.sederholm@hsc.utah.edu

**Keywords:** Parkinson’s disease, medication errors, drug-related side effects and adverse reactions, antipsychotic agents

## Abstract

The purpose of this study was to evaluate the rate at which potentially inappropriate medications were administered for patients diagnosed with Parkinson’s disease (PD). This is a single-center, retrospective, case cohort study with data collected at an academic medical center between January 2010 and December 2013. Participants included all adult patients with admission diagnosis codes for PD. Included patients were screened for administrations of 27 potentially inappropriate medications and two potentially appropriate medications to be used for comparison. There were 1736 patients who met inclusion criteria with 175 documented administrations of potentially inappropriate medications to 77 patients. Patients who received potentially inappropriate medications had a longer mean duration of stay than the baseline population of PD patients (3.3 days vs. 1.9 days, *p*-value < 0.001). Despite recommendations to avoid certain medications in PD patients, a substantial number of administrations still occurred. The use of these medications can have clinical implications and our findings demonstrate increases in duration of stay. The findings from this study can assist in developing technological alerts to reduce inappropriate prescribing to PD patients. Larger prospective studies are warranted to further investigate the administration of inappropriate medications to patients diagnosed with PD.

## 1. Introduction

Parkinson’s disease (PD) is a progressive neurological disorder that currently affects approximately 2% of people over 80 years of age. As of 2011, over 4.1 million individuals over the age of 50 are affected by PD, and that number is expected to double by 2030 [1,2]. Patients with PD are typically managed by neurologists and movement disorder specialists in the outpatient setting. When PD patients are admitted to acute care settings, they transition to non-specialist providers and complications can arise when medications are not managed appropriately [1].

The initial treatment of PD patients upon admission to hospitals is complicated due to unique factors (e.g., medication management, psychiatric problems, falls), which can have serious implications on the health of patients [3]. A diagnosis of PD is associated with longer hospitalization times and increased hospitalization rates [1]. The quality of care also varies depending on whether the patient is being admitted to a neurology service versus another service. Additionally, the care that the patient receives is dependent on whether the patient is admitted with Parkinsonian features but without a formal diagnosis of PD, the patient has PD but is hospitalized for unrelated problems, or the patient is hospitalized for PD complications [2].

One critical problem with hospitalized PD patients involves the ordering of potentially inappropriate medications. A number of common inpatient medications can have serious negative impacts on patients with PD. Examples include certain antiemetic medications, antipsychotic medications, pain medications, and others [4]. A survey of National Parkinson Foundation Centers indicated that 71% of centers were not confident that hospital professionals knew that certain antiemetic medications were contraindicated, and 80% were not confident that hospital professionals knew which antipsychotics would least likely exacerbate PD symptoms [1].

The goal of this study was to evaluate the rate at which potentially inappropriate medications were administered to inpatients diagnosed with PD and to determine if there was an association between these administrations and hospital length of stay.

## 2. Materials and Methods 

This was a single-center, retrospective, observational study at a 488-bed academic medical center. The study included patients greater than or equal to 18 years of age with PD who were admitted as inpatients between 1 January 2010 and 31 December 2013. Patients with PD were defined as those having a primary and/or secondary discharge International Statistical Classification of Diseases and Related Health Problems, Ninth Revision, (ICD-9) diagnosis code related to PD (see Table A1 in Appendix A). No exclusion criteria were used. Patients meeting inclusion criteria are referred to in this study as the “Baseline PD Population.” Patients were then filtered for those who received at least one inpatient administration of a potentially inappropriate medication. The following were considered potentially inappropriate medications: aripiprazole, asenapine, chlorpheniramine, chlorpromazine, clozapine, cyclizine, droperidol, fluphenazine, haloperidol, iloperidone, lithium, loxapine, lurasidone, meperidine (if patient concurrently taking a monoamine oxidase B inhibitor), metoclopramide, olanzapine, paliperidone, perphenazine, pimozide, prochlorperazine, promethazine, risperidone, thioridazine, thiothixene, trifluoperazine, valproate, and ziprasidone. 

The primary objective was to assess the rate at which patients with PD received potentially inappropriate medications. This rate was measured using: (1) total number of patients receiving potentially inappropriate medication; and (2) total number of administrations of potentially inappropriate medications. These numbers were then analyzed and summarized in raw totals and also normalized by number of baseline PD patients. The secondary objective was to determine if there was an association between administrations of potentially inappropriate medications and hospital length of stay. This analysis was done by comparing the hospital length of stay amongst three different population pairs: (1) comparison was made between the baseline PD population and those administered a potentially inappropriate medication; (2) amongst patients administered an antipsychotic, comparison was made between those administered quetiapine (an antipsychotic that is not contraindicated in PD patients) and those administered a potentially inappropriate antipsychotic; and (3) amongst patients administered an antiemetic, comparison was made between those administered trimethobenzamide (an antiemetic that is not contraindicated in PD patients) and those administered a potentially inappropriate antiemetic. A potentially inappropriate antipsychotic was defined as one or more inpatient administrations of aripiprazole, chlorpromazine, haloperidol, lithium, olanzapine, or ziprasidone. A potentially inappropriate antiemetic was defined as one or more inpatient administrations of droperidol, metoclopramide, prochlorperazine, or promethazine. An electronic database query was used to identify patients, collect patient demographic information, and collect medication administration outcomes. Baseline demographic and clinical characteristics were summarized using means, frequencies, and percentages. Statistical analyses were performed using SAS V9.3 (Cary, NC) and Stata 13 (College Station, TX) assuming two-sided alpha of 0.05. All study activities were reviewed and approved by the health-system’s institutional review board and deemed exempt.

## 3. Results

**Figure 1:**
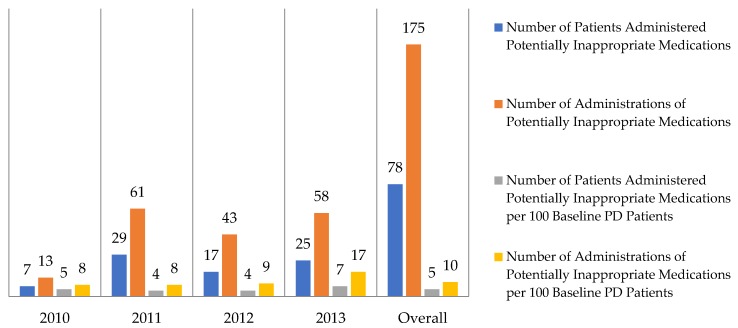
Rates of potentially inappropriate medication administration in hospitalized patients with Parkinson’s disease (PD).

There were 1736 patients who met inclusion criteria and who functioned as the baseline PD population. During the four-year study period, there were 175 documented administrations of potentially inappropriate medications to 78 patients. The rates at which patients with Parkinson’s disease received potentially inappropriate medications are summarized in Figure 1. The baseline PD population had a mean age of 52.4 years and patients administered potentially inappropriate medications had a mean age of 56.1 years. The most commonly administered potentially inappropriate medications included droperidol (16% of all potentially inappropriate administrations), aripiprazole (15%), and promethazine (14%). All potentially inappropriate medications that were administered are summarized in Table 1. In regard to the secondary outcome, patients administered potentially inappropriate medications were admitted for 1.4 days longer than the baseline PD population (*p*-value < 0.001). Patients administered potentially inappropriate antipsychotics and antiemetics also had a longer hospital length of stay than their respective comparators, but this difference was not statistically significant. These secondary outcomes are summarized in Table 2. Of patients who received inpatient administrations of potentially inappropriate antipsychotics, no patients were subsequently transitioned to inpatient administrations of quetiapine.

## 4. Discussion

In this analysis of PD patients, we found two significant findings. First, there was a significant number of PD patients administered inappropriate medications within a four-year timespan. Second, patients who were administered inappropriate medications had a statistically significant longer duration of hospital stay compared to the baseline PD population.

Parkinson’s disease has the potential to be overlooked when assessing potential medication errors and contraindications. At our academic medication center, despite long-standing use of electronic medication order entry and verification, there are no alert systems that warn healthcare providers when they are attempting to order inappropriate medications for PD patients. The use of alert systems could potentially decrease complications in PD patients. Examples of potential interventions include: Clinical decision support for antipsychotic or antiemetic medications in patients with PD, electronic alerts or warnings when providers attempt to order these potentially inappropriate medications in patients with PD, and the requirement for a neurological consultation when antipsychotics other than quetiapine or pimavanserin are ordered in patients with PD. Electronic medical records and computerized order entry are technologies purported to promote patient safety, but these systems can fail subsets of the population. In order to assess the effectiveness of a future alert system, and in order to make improvements to the system in the future, it is critical to know the rate at which inappropriate medications have been, and are currently being, ordered for PD patients. This study was designed to support future development and implementation of these types of systems.

In addition to inappropriate medication ordering, other difficulties arise in the treatment of PD inpatients. One concern is that patients’ outpatient PD medications can be restarted inappropriately at the hospital (e.g., wrong doses, timing, etc.); this can lead to alterations in mental status. Altered mental status can lead providers to order antipsychotic medications, such as quetiapine. These medications may not have been required if outpatient PD medications were restarted more appropriately. As a result, the rate of antipsychotic use was assessed and shown to have significantly high rates of inappropriate antipsychotic administrations compared to a preferred antipsychotic, quetiapine. Finally, the use of inappropriate antiemetic medications represented 46% of all inappropriate administrations.

Limitations should be noted when interpreting study findings. First, with all observational studies, there is a possibility of unmeasured variables affecting causal inferences. One potential confounding variable to note is patient comorbid conditions which was not an outcome included in this study. Differences in comorbid conditions may have impacted duration of hospital stay. Second, this was a single-center study performed in an academic medical center and the practice pattern may not be applicable to hospitals serving small numbers of PD patients. Third, we included medications that are not expressly contraindicated in PD patients, and we also included PD subsets. This conservative approach may have diminished the effect of medications on hospital length of stay and thus understated the impact of more clinically important contraindicated medications (e.g., haloperidol) in PD patients. Fourth, a patient’s baseline outpatient medication regimen can influence inpatient medication decisions, but baseline outpatient medication regimens were not an outcome included in this study.

In conclusion, at an academic medical center, we observed a high prevalence of administrations of potentially inappropriate medications for patients with Parkinson’s disease (175 administrations over four years). These administrations may cause an extended duration of hospital stay as patients receiving these potentially inappropriate medications were observed to have an increased duration of hospital stay compared to the baseline PD population (3.3 days versus 1.9 days, *p*-value < 0.001). Larger studies are needed to validate these results and interventions, such as clinical decision support, are warranted to help improve medication use in this population.

## Figures and Tables

**Table 1:** Potentially inappropriate medications administered.

Medications Administered, n (% of Total Potentially Inappropriate Administrations)	Number of Administrations (n = 175)
Droperidol	27 (15)
Aripiprazole	26 (15)
Promethazine	25 (14)
Prochlorperazine	23 (13)
Meperidine *	20 (11)
Olanzapine	19 (11)
Lithium	13 (7)
Ziprasidone	10 (6)
Haloperidol	5 (3)
Metoclopramide	5 (3)
Chlorpromazine	2 (1)

* Only included in analysis if patient was concurrently being administered a monoamine oxidase B inhibitor.

**Table 2:** Patient characteristics and outcomes.

	Patients Administered PI Medications	Baseline PD Population	Patients Administered PI Antipsychotic ±	Patients Administered Quetiapine	Patients Administered PI Antiemetic †	Patients Administered TMBA
Age, mean	56.1	52.4	51.6	66.6	56.4	64.2
Patients, n	78	1736	12	5	70	105
Administrations, n	175	N/A	75	15	80	199
Days of hospitalization, n	3.3	1.9	7.0	6.2	2.6	2.1
*P*-Value	< 0.001		0.867		0.237	

Abbreviations: N/A = not applicable; PD = Parkinson’s disease; PI = Potentially inappropriate; TMBA = Trimethobenzamide. ± Defined as one or more inpatient administrations of aripiprazole, chlorpromazine, haloperidol, lithium, olanzapine, or ziprasidone. † Defined as one or more inpatient administrations of droperidol, metoclopramide, prochlorperazine, or promethazine.

## Appendix A

**Table A1:** Parkinson’s disease admission diagnosis codes [5].

ICD-9 Diagnosis Codes
331.9 Cerebral degeneration, unspecified
332.0 Paralysis agitans
332.1 Secondary parkinsonism
333.0 Other degenerative diseases of the basal ganglia
333.1 Essential and other specified forms of tremor
781.0 Abnormal involuntary movements
781.3 Lack of coordination

Abbreviations: ICD-9 = International Statistical Classification of Diseases and Related Health Problems, Ninth Revision.
